# Evaluation of imputation performance based on the single nucleotide polymorphism panel density and the reference population size in Korean native chicken

**DOI:** 10.5713/ab.24.0815

**Published:** 2025-04-04

**Authors:** Minjun Kim, Hyo Jun Choo, Sunghyun Cho, Doo Ho Lee, Jun Heon Lee, Dongwon Seo

**Affiliations:** 1 Department of Animal Science, Chungnam National University, Daejeon, Korea; 2 Poultry Research Institute, National Institute of Animal Science, Rural Development Administration, Pyeongchang, Korea; 3 Research and Development Center, Insilicogen Inc., Yongin, Korea; 4 Quantomic Research & Solution, Daejeon, Korea; 5 Research Institute TNT Research Company, Jeonju, Korea

**Keywords:** Imputation, Korean Native Chicken, Reference Population Size, Single Nucleotide Polymorphism (SNP) Density

## Abstract

**Objective:**

This study aimed to identify the optimal single nucleotide polymorphism (SNP) panel density for accurate imputation in the Korean native chicken (KNC) and Yeonsan Ogye (YO) populations. The primary focus was on evaluating how the reference population size and SNP density influence imputation performance and accuracy.

**Methods:**

Data were collected from five purebred lines of KNC and the YO population, comprising a total of 256 KNC and 199 YO chickens. Imputed genotype ratio and accuracy were evaluated across various scenarios using SNP densities of 2.5K, 5K, 10K, and 50K in both populations. Additionally, for the YO dataset, reference population sizes of 50, 100, and 150 were analyzed to assess their impact on imputation outcomes.

**Results:**

Higher SNP densities notably improved imputation performance. Specifically, when SNP panel density reached 10K or greater, the ratio of imputed SNPs exceeded 70% and the accuracy increased substantially, regardless of the reference population size. However, imputation efficiency decreased markedly when either the reference or test population size was around 50 individuals.

**Conclusion:**

A test SNP density of at least 10K was determined to be essential for accurate genotype imputation. Additionally, imputation efficiency was observed to decline when either the reference or test population included around 50 individuals. These findings provide important data that can guide the genetic improvement of indigenous livestock populations.

## INTRODUCTION

Recent consumer trends have shown a preference for quality over quantity in meat consumption, highlighting the importance of improving indigenous livestock breeds known for superior meat quality and flavor. The Korean native chicken (KNC) is an indigenous breed of Korea, renowned for its superior taste compared to commercial broilers. Numerous studies have provided scientific evidence supporting the superior flavor profile of KNC meat. Especially, the meat of KNC broiler contains a higher amount of taste-active compound which makes umami taste and enhanced flavor than commercial broiler [1–3]. However, KNC population has undergone inferior growth performance compared to commercial chicken breeds [4,5]. Indigenous livestock breeds generally show less productivity and this makes it difficult to utilize the breed for commercial usage [6,7]. Therefore, further genetic improvement is necessary to overcome the disadvantages of low growth rates and egg production while maintaining the benefit of superior meat quality. Genetic improvement in livestock is a time-intensive process; thus, to accelerate the breeding progress, it is imperative to implement an efficient breeding system utilizing genomic information [8,9].

Genomic selection is typically performed based on single nucleotide polymorphism (SNP) chip genotyping. High-density SNP panels enable accurate genomic prediction and genome-wide association studies (GWAS); however, they are associated with high genotyping costs. Imputation technology, which infers the genotypes of high-density SNP panels from those of lower-density SNP panels based on the population linkage disequilibrium structure, offers a cost-effective solution. Imputation predicts the ungenotyped SNPs of animals using haplotypes present in the population [10]. In chickens, low- or medium-density SNP chip genotypes can be used to infer genotypes for the high-density Affymetrix 600K SNP chip. This approach allows the efficient acquisition of genotypes for large numbers of individuals and is well-suited for population studies, including genomic selection and GWAS [9]. By utilizing low-density panel genotyping, this method reduces data production costs, allowing for the accumulation of a larger dataset [11]. Imputation offers an additional advantage, as it facilitates the integration of genotype data across SNP panels with different densities and marker combinations. Thus, even if the marker panels of current and future genotype data differ, they can be integrated to create a cohesive dataset. Employing a large reference population for imputation increases the precision of genomic selection and reliable GWAS results by providing a more extensive and varied set of genetic information. This approach is not only applicable to chickens but also extends to other species, such as cattle and sheep, where imputation plays a crucial role in genomic studies and breeding programs [12–16].

Several factors significantly affect the efficiency of genotype imputation, with marker density and sample size being particularly crucial. Higher marker density improves imputation accuracy by providing more data and reducing gaps in genomic information, leading to more precise genotype predictions. Similarly, larger sample sizes in the reference panel contribute to better imputation performance by capturing a wider range of genetic diversity and reducing the likelihood of missing variants. These elements collectively increase the robustness and reliability of imputed genotypes [10].

The LD of the target population also significantly affects the efficiency of imputation. Genotype prediction relies on the LD structure of the population, and the prediction model performed more accurately when accounting for LD decay [17]. Additionally, if a population has experienced bottlenecks or strong selection pressure, these factors can contribute to the formation of strong LD patterns, which may influence imputation efficiency. The more similar the reference panel is to the study population, the more aligned their LD structures will be, resulting in higher imputation accuracy [18]. The objective of this study was to evaluate the efficiency of imputation in a single or multi-population datasets and to assess how the size of the reference population and SNP density influence imputation efficiency. We examined various imputation scenarios to identify the minimum marker set capable of representing the haplotype structure of the KNC genome.

## MATERIALS AND METHODS

### Animal care

All samples used in this research were collected according to guidelines issued by the Institutional Animal Care and Use Committee of Chungnam National University, who approved this study (approval no. CNU-00486).

### Animals and genotypes

We used five lines of the KNC population (red-brown, yellow-brown, white, black, and gray) and the Yeonsan ogye (YO) chicken population. First, we genotyped 208 KNCs (59 red-brown, 39 yellow-brown, 40 white, 30 black, and 40 gray-brown; raised at the National Institute of Animal Science farm), and 189 YO chickens (raised at the Jisan farm) with a 600K chicken SNP array (Affymetrix, Santa Clara, CA, USA). Additionally, whole-genome sequence (WGS) data for 48 KNCs (across the five lines) and 10 YO chickens were obtained from the Korean National Agricultural Biotechnology Information Center (https://nabic.rda.go.kr/). Raw sequence reads were mapped to the Gallus_gallus_5.0 reference genome, and variants were called using the Genome Analysis Toolkit v4.1.4.1. To create the 600K panel dataset from the WGS variant data, an SNP list was generated based on chromosome positions using the Affymetrix 600K array batch file; the variants included in the SNP list were extracted from all genotype data. The 600K data for 48 KNC and 10 YO extracted from the WGS were added, resulting in a final dataset comprising 256 KNC (69 red-brown, 47 yellow-brown, 50 white, 40 black, and 50 gray-brown) and 199 YO chickens for analysis. The SNPs in the 600K panel genotype data were filtered using PLINK v1.9, according to the following criteria: genotype call rate > 0.9, Hardy–Weinberg equilibrium p<10^−4^, and minor allele frequency >0.01. Because we constructed two datasets, one from the YO population and the other from the KNC population, genotype quality control was applied separately for each dataset, resulting in 548,646 and 486,759 filtered SNPs for the 600K panel data, respectively.

### Imputation scenarios

Simulation tests were conducted separately for the single and multi-population datasets. Imputation scenarios within a single population utilized YO data. To assess the impact of reference and test population sizes on imputation performance, the YO population was randomly divided into three datasets (1 to 3) with varying reference:test ratios: 1) 50 reference birds:149 test birds, 2) 100 reference birds:99 test birds, and 3) 150 reference birds:49 test birds. To compare imputation results according to SNP density, test population data were selected at densities of 2.5K (2,486 SNPs), 5K (4,972 SNPs), 10K (9,944 SNPs), and 50K (49,720 SNPs). Markers were selected by evenly spacing them across each chromosome to reduce the density from 600K.

**Figure 1:**
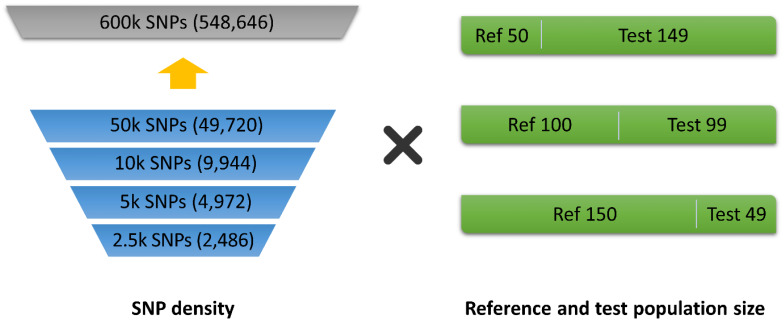
Experimental design for imputation based on single-nucleotide polymorphisms (SNP) density and the size of the reference and test populations.

Data from the five KNC lines were used to simulate imputation using the multi-population genotype data. The population was divided into two groups of 128 birds each to form the reference and test groups. This dataset was then used to evaluate imputation performance according to SNP panel density. The method used to reduce SNP density in the test population was the same as that applied to the YO data. The experimental design is illustrated in Figure 1, and the imputation scenarios are summarized in Table 1.

### Imputation methods and evaluation

Haplotype phasing of reference and test data was implemented using Eagle version 2.4.1 software. The imputation of test data based on the reference haplotypes was performed using Minimac3 software. Both the phasing and imputation were implemented with default parameter settings. Imputation performance was evaluated by calculating the ratio of the number of predicted genotypes to the number of genotypes in the 600K SNPs (548,646 SNPs), and imputation accuracy was assessed by calculating Rsq as the squared correlation between the imputed SNPs and the true unobserved genotypes (https://genome.sph.umich.edu/wiki/Minimac3_Info_File), and by calculating the original accuracy as the Pearson correlation coefficient between the imputed and true genotypes. For the simulation tests using the multi-population, multi-dimensional scatter (MDS) plots based on the imputed genotype data were generated using PLINK v1.9 with mds-plot option and R v3.6.3 software (R Core Team, Vienna, Austria) to examine changes in the explanatory power of genetic differences between populations under different imputation conditions.

## RESULTS

### Imputation performance of low- to high-density panels in the single population dataset

**Figure 2:**
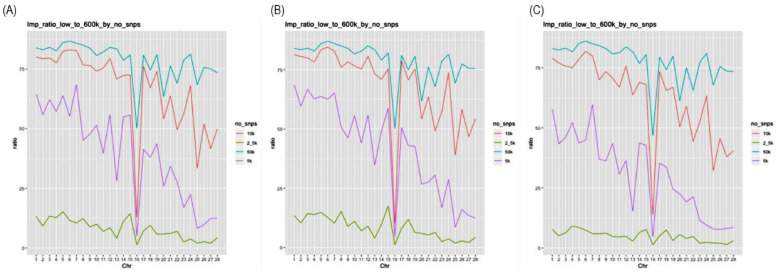
Imputation ratio of genotypes imputed from low-density single-nucleotide polymorphisms to a 600K density in the single population of Yeonsan ogye chickens for reference population sizes of (A) 50, (B) 100, and (C) 150 individuals. No_snps, number of single nucleotide polymorphism; chr, chromosome.

The imputation performance from a low-density panel to a 600K density panel within a single population was evaluated based on the test panel density (2.5K, 5K, 10K, or 50K) and reference population size (50, 100, or 150). As shown in Table 2, for the scenario Single_2.5K_Ref50, 31,638 SNPs were imputed, resulting in an imputation ratio of 5.77%. In contrast, when the reference population size was 100 and 150 for the same density level (e.g., scenarios Single_2.5K_Ref100 and Single_2.5K_Ref150), the numbers of imputed variants increased to 59,114 (10.77%) and 55,588 (10.13%), respectively. The difference in imputation ratio between scenarios for the 2.5K density reached up to 5.00%. For 5K density, 227,704 SNPs were imputed in the Single_5K_Ref50 scenario, with an imputation ratio of 41.50%. In the Single_5K_Ref100 and Single_5K_Ref150 scenarios, 301,678 SNPs (54.99%) and 284,842 SNPs (51.92%) were imputed, respectively. The difference in the imputation ratio between scenarios for 5K density reached up to 13.44%, which was the highest among all density levels. For 10K density, as the reference population size increased from 50 to 100 to 150, the number of imputed SNPs varied from 388,055 (70.73%) to 412,850 (75.25%) and 405,076 (74.01%), respectively. The maximum difference in imputation ratio for the 10K density scenarios was 4.52%. Lastly, for 50K density, the number of imputed SNPs for reference population sizes of 50, 100, and 150 were 396,958 (72.35%), 403,360 (73.52%), and 402,012 (73.27%), respectively; the differences in the ratios among these scenarios was up to 1.17%. The scenario with the best imputation performance in the single population test was Single_50K_Ref100, where the reference and test populations were similar in size and the highest density was used. Additionally, the imputation ratio for chromosome 16 was remarkably lower than those for other chromosomes (Figure 2).

### Imputation performance of low- to high-density panels in the multi-population dataset

**Figure 3:**
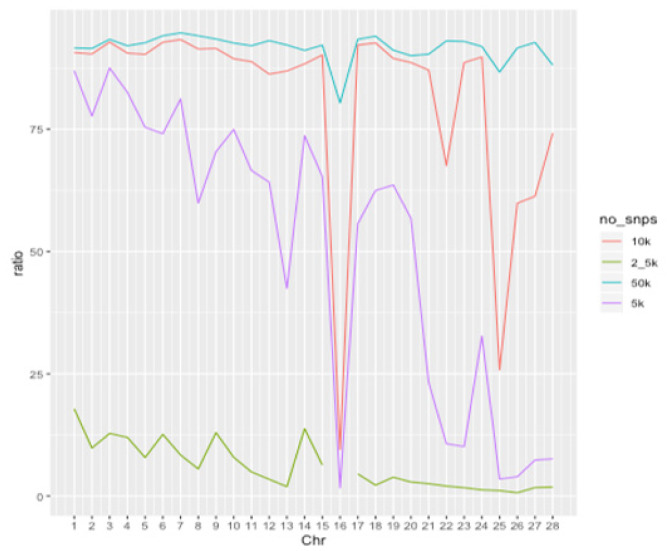
Imputation ratio of genotypes imputed from low-density single-nucleotide polymorphisms (SNPs) to a 600K density in the five Korean native chicken lines. No_snps, number of SNPs; chr, chromosome.

When the reference and test populations were both derived from multi-population datasets of the five KNC lines, the number and ratio of imputed SNPs for the 600K density panel varied according to the test panel density (2.5K, 5K, 10K, or 50K), as follows. As shown in Table 2 and Figure 3, for the Multi_2.5K scenario, 43,272 variants were imputed, representing 8.89% of the 600K panel SNPs. In contrast, in the Multi_5K scenario, 227,704 SNPs were imputed, yielding a ratio of 49.47%. Although the panel density of the test dataset doubled, the number of imputed variants increased by a factor of 5.56. For the Multi_10K and Multi_50K scenarios, with panel densities of 10K and 50K, 356,940 and 356,258 SNPs were imputed, with ratios of 73.33% and 73.19%, respectively. Thus, increasing the panel density from 5K to 10K resulted in a 1.48-fold improvement in imputation efficiency, whereas increasing the density from 10K to 50K did not yield further improvements in efficiency. Similar to the case of a single population, chromosome 16 had a notably lower imputation ratio than the other chromosomes; the Multi_50K scenario was the only scenario in which the ratio exceeded 75%.

### Imputation accuracy of low- to high-density panels in the single population dataset

**Figure 4:**
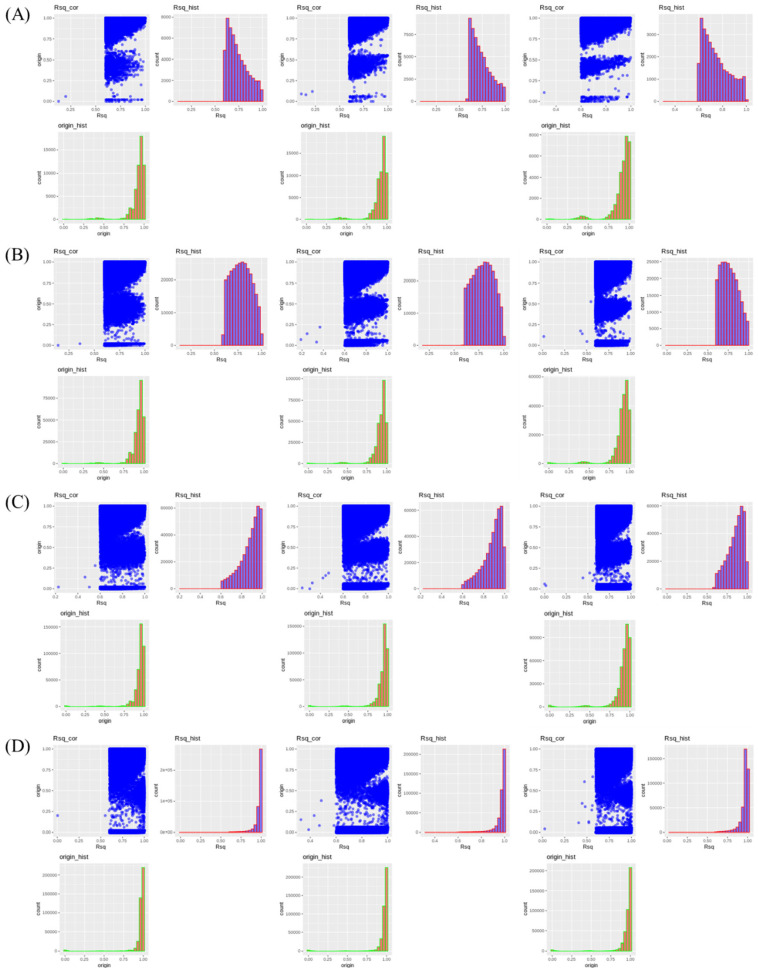
Accuracy differences for genotypes imputed to a 600K density in the single population of Yeonsan ogye, according to reference population size and single-nucleotide polymorphism density. (A–D) Imputation accuracy from (A) 2.5K, (B) 5K, (C) 10K, or (D) 50K to 600K, for reference population sizes of (left) 50, (middle) 100, and (right) 150 individuals.

The imputation accuracy for the single population dataset is presented in Figure 4. No noticeable changes in accuracy were observed with varying reference population sizes under any test panel density conditions. As the test panel density increased, Rsq values approached 1. Notably, the distribution was skewed to the left for the 2.5K and 5K densities, but skewed to the right for the 10K and 50K densities, with most SNPs having an Rsq value of 1 at the 50K density. The distribution of original accuracy values also converged towards 1 with increasing density. The scenarios with 50K density showed the highest original accuracy, with most SNPs having an original accuracy of 1.

### Imputation accuracy of low- to high-density panels in the multi-population dataset

**Figure 5:**
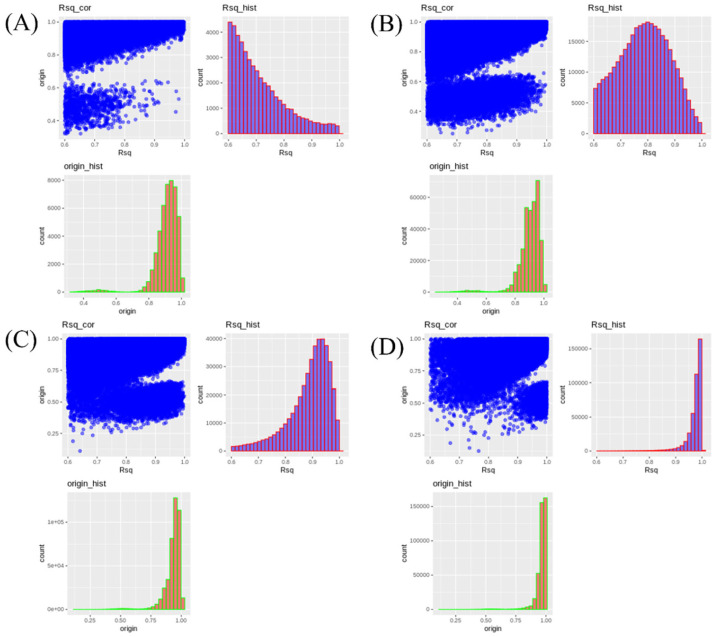
Accuracy differences of genotypes imputed to 600K according to single-nucleotide polymorphism density in the five Korean native chicken lines. (A–D) Imputation accuracy corresponding to (A) 2.5K, (B) 5K, (C) 10K, and (D) 50K density datasets.

The imputation accuracy for the multi-population dataset is presented in Figure 5. Both Rsq values and original accuracy gradually increased to approach 1 as the panel density increased. The Rsq distribution was skewed to the left at 2.5K density, but shifted to the right at 10K and 50K densities, with most Rsq values close to 1 as the density approached 50K. The average Rsq value for imputation scenarios was approximately 0.741 for Multi_2.5K and about 0.970 for Multi_50K (Table 3). These results indicate that a panel density of 50K or higher is necessary to achieve a theoretical accuracy above 0.9. The original accuracy was approximately 0.927 for Multi_2.5K and about 0.967 for Multi_50K. These results indicate that as the SNP density of the test panel increased, both the Rsq value and the original accuracy increased, and the difference between these two accuracy parameters decreased (Table 3).

### Multi-dimensional scatter plots based on imputed and original genotypes of the multi-population dataset

**Figure 6:**
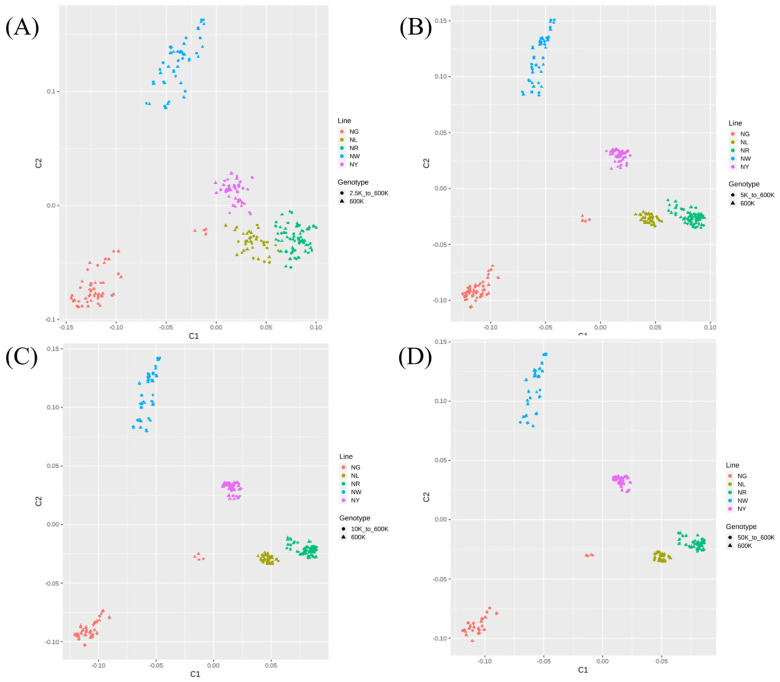
Principal component analysis results based on genotypes imputed to 600K using different single-nucleotide polymorphism densities in datasets from five Korean native chicken (KNC) lines. (A–D) Results obtained using (A) 2.5K, (B) 5K, (C) 10K, and (D) 50K datasets. NG, KNC grey-brown; NL, KNC black; NR, KNC red-brown; NW, KNC white; NY, KNC yellow-brown.

The MDS analysis results (Figure 6) were used to assess whether the imputed genotypes from the multi-population dataset accurately reflected the genetic characteristics of each line and highlighted their differences. Across all scenarios, clusters of each line were observed to be distinct and non-overlapping on the MDS plots. Importantly, when the test panel density was 5K or higher, the clustering of each group became notably more defined. At densities of 5K or higher, the positions of samples based on the actual 600K genotype and the imputed genotype tended to align closely.

## DISCUSSION

This study was conducted to develop a low-density SNP panel optimized for accurate imputation to a high-density chicken SNP panel. Given that the sample size of the reference panel is a critical factor contributing to imputation performance, simulations were performed to evaluate whether acceptable results could be obtained under varying conditions by adjusting the sample sizes of the reference and test populations during genotype imputation. Simulation tests using a single population revealed superior imputation performance when the reference population was larger than the test population, compared to the opposite scenario. Furthermore, the highest imputation performance was observed when the reference population size was 100. This trend was observed consistently, even as the test panel density increased. With the exception of the scenario using the 5K density panel in the single population dataset, the difference in imputation ratio according to the reference population size was less than 5%. Moreover, no meaningful changes in accuracy were observed based on the size of the reference population under any test panel density condition. Previous studies have also reported that the reference population size is a major factor contributing to imputation efficiency. The relatively small sample size used in this study compared to other studies may explain the minimal differences in imputation and accuracy observed with varying reference population sizes [19–21].

We performed imputation tests to determine the optimal number of markers for a low-density SNP panel to achieve stable imputation performance. In both single and multi-populations, a test panel density of 10K or higher was found to predict over 70% of the missing genotypes, regardless of the reference population size. Previous studies using Rhode Island and Leghorn chicken breeds for imputation tests for 600K chip genotype also reported high, stable accuracy when the test panel density exceeded 10K [22]. In contrast, in the multi-population, imputation accuracy increased at higher test panel densities, with the highest accuracy observed at 50K density. Similarly, previous studies using datasets from mixed pig populations for both the reference and test populations showed that imputation accuracy improved as the test panel density increased [11]. We confirmed through MDS analysis based on genotype data from multi-populations that a test panel with at least 5K SNP density is required to perform clear breed differentiation using imputed 600K genotype data. Various studies have employed different approaches to select informative markers for designing low-density SNP panel. These methods include selecting markers at equal physical distances within chromosomes, prioritizing markers with high minor allele frequency, and applying LD pruning to retain only representative markers from LD blocks. A combination of these strategies is often utilized to optimize SNP selection [22–25]. Previous research has reported that higher accuracy of genomic estimated breeding values (GEBVs) predicted using imputed genotypes from high-density SNP panels [11]. This improvement in GEBVs is due to high imputation accuracy. To enhance imputation accuracy, it has been recommended that the reference population size be increased by including genotypes from other populations as references [19]. Therefore, in small local populations such as the KNC, performing imputation and genomic prediction using genotype data from genetically related populations could strategically lead to higher accuracy.

Even in scenarios using high test panel densities, the SNPs on chromosome 16 consistently showed a lower genotype imputation ratio, likely for several reasons. In chickens, chromosome 16 is a microchromosome with extremely polymorphic and repetitive sequence regions. Furthermore, this microchromosome shows high recombination rate. It results in a lower number of variants included in the 600K SNP array. imputation accuracy tends to be lower for microchromosomes than for macrochromosomes due to their unique LD structure and increased recombination frequency. Specifically, microchromosomes require a denser marker distribution for improvement of imputation accuracy, as lower marker coverage results in fragmented haplotypes and reduced genotype predictability [22,26]. Clarifying the sequences of poorly characterized genomic regions, including the major histocompatibility complex region, selecting markers that enable more precise detection of LD, and utilizing population-specific markers in SNP panels are also effective strategies.

This condition likely contributed to the lower imputation ratio observed for chromosome 16 in this study. The LD scores of KNC and YO showed a small difference, ranging from 0.19 to 0.22 when using markers within 300 kb [27]. Unlike some other indigenous breeds, the LD scores of KNC and YO exhibited only a minor difference of approximately 0.1 compared to commercial chicken breeds [27,28]. Future studies could utilize lines with particularly low or high LD to investigate their impact on imputation performance, providing valuable foundational data. This study identified the optimal SNP panel density for imputation to the 600K SNP panel in the KNC population. These findings provide valuable information for SNP chip development. As more genetic information is obtained from a population, a better understanding of that population can be obtained, which enhances the efficiency of genomic predictions and GWAS. It is essential to use an optimally designed SNP chip to accumulate genotypic information within a limited budget for achieving highly accurate imputation, GEBV estimation, and reliable quantitative trait loci. In the case of KNC and YO populations, leveraging their superior meat quality traits for industrial applications requires the implementation of genomic prediction-based breeding strategies to improve growth performance traits. The application of genomic prediction to body weight traits in chickens has previously been demonstrated [8].

The markers included in the SNP chip should meet the minimum number required to achieve high-density genotypes through imputation. Indeed, multiple studies have emphasized the need to determine the optimal number of markers and sample sizes through imputation tests. For example, in previous studies, various imputation scenarios were tested to determine the optimal marker combination when developing SNP chips for chickens [22], and imputation was effectively utilized to achieve highly accurate genotype data during SNP panel development in dairy cattle [29]. These studies highlighted the essential role of imputation tests in SNP chip development, and that the results of the present study represent crucial foundational data for developing a customized SNP chip for populations with distinct regional characteristics. While this study focused on KNC, the approach and insights gained can be broadly applied to other livestock species, such as cattle and sheep, to enhance genomic selection and breeding programs. Additionally, we acknowledge that the comparatively small sample size used in this study may limit the generalizability of our conclusions. To address this limitation, future research will focus on expanding the reference population by incorporating genetically related indigenous chicken populations [30]. Despite these constraints this approach will ultimately enhance the accuracy of genomic selection and support the design of efficient, cost-effective livestock improvement programs.

## CONCLUSION

In this study, we identified the optimal SNP panel density for imputation to a 600K panel in the KNC population. We also investigated the appropriate ratio between reference and test populations. In both single and multi-populations, a test panel density of at least 10K was found to be necessary for the accurate estimation of imputed genotypes. Furthermore, we observed that imputation efficiency was notably reduced when either the reference or test population comprised fewer than 50 individuals. The findings of this study represent valuable data for the improvement of indigenous livestock populations.

## Figures and Tables

**Table 1:** Imputation scenarios with 600K single-nucleotide polymorphism genotypes of different densities and reference population size in single and multi-populations

Scenario	Number of reference animal	Number of imputed animal	Density of panel of imputed animal	Density of reference panel	Description of population
Single^1)^_2.5K_Ref50	50	150	2,486	548,646	YO
Single_2.5K_Ref100	100	100	2,486	548,646	YO
Single_2.5K_Ref150	150	50	2,486	548,646	YO
Single_5K_Ref50	50	150	4,972	548,646	YO
Single_5K_Ref100	100	100	4,972	548,646	YO
Single_5K_Ref150	150	50	4,972	548,646	YO
Single_10K_Ref50	50	150	9,944	548,646	YO
Single_10K_Ref100	100	100	9,944	548,646	YO
Single_10K_Ref150	150	50	9,944	548,646	YO
Single_50K_Ref50	50	150	49,720	548,646	YO
Single_50K_Ref100	100	100	49,720	548,646	YO
Single_50K_Ref150	150	50	49,720	548,646	YO
Multi^2)^_2.5K	128	128	2,486	548,646	NR+NY+NG+NL+NW
Multi_5K	128	128	4,972	548,646	NR+NY+NG+NL+NW
Multi_10K	128	128	9,944	548,646	NR+NY+NG+NL+NW
Multi_50K	128	128	49,720	548,646	NR+NY+NG+NL+NW

1) Single population dataset.

2) Multi population dataset.

YO, Yeonsan ogye; NR, Korean native chicken Red-brown; NY, Korean native chicken Yellow-brown; NG, Korean native chicken Grey-brown; NL, Korean native chicken Black; NW, Korean native chicken White.

**Table 2:** Number of imputed variants and ratio of imputed variants by the number of variants in the 600K single-nucleotide polymorphism panel

Scenario	Number of SNPs	Number of imputed SNPs	Ratio of imputed SNPs to 600K SNPs (%)
Single^1)^_2.5K_Ref50	2,406	31,638	5.77
Single_2.5K_Ref100	2,406	59,114	10.77
Single_2.5K_Ref150	2,406	55,588	10.13
Single_5K_Ref50	4,972	227,704	41.50
Single_5K_Ref100	4,972	301,678	54.99
Single_5K_Ref150	4,972	284,842	51.92
Single_10K_Ref50	9,944	388,055	70.73
Single_10K_Ref100	9,944	412,850	75.25
Single_10K_Ref150	9,944	406,076	74.01
Single_50K_Ref50	49,720	396,958	72.35
Single_50K_Ref100	49,720	403,360	73.52
Single_50K_Ref150	49,720	402,012	73.27
Multi^2)^_2.5K	2,406	43,272	8.89
Multi_5K	4,972	240,799	49.47
Multi_10K	9,944	356,940	73.33
Multi_50K	49,720	356,258	73.19

1) Single population dataset.

2) Multi population dataset.

**Table 3:** Comparison of imputation accuracy to 600K data based on differences in the density of datasets from the five Korean native chicken lines

Scenario	R-squared	Original accuracy^1)^
Multi_2.5K	0.740909	0.926893
Multi_5K	0.78684	0.927757
Multi_10K	0.878449	0.943006
Multi_50K	0.969823	0.966509

1) Pearson correlation coefficient between the imputed and true genotypes.
